# Antinociceptive and Antibacterial Properties of Anthocyanins and Flavonols from Fruits of Black and Non-Black Mulberries

**DOI:** 10.3390/molecules23010004

**Published:** 2017-12-21

**Authors:** Hu Chen, Wansha Yu, Guo Chen, Shuai Meng, Zhonghuai Xiang, Ningjia He

**Affiliations:** State Key Laboratory of Silkworm Genome Biology, Southwest University, Beibei, Chongqing 400715, China; c9249@email.swu.edu.cn (H.C.); summer92@email.swu.edu.cn (W.Y.); chenguo1992@email.swu.edu.cn (G.C.); xndxms@163.com (S.M.); xbxzh@swu.edu.cn (Z.X.)

**Keywords:** anthocyanins, antibacterial, antinociceptive, flavonols, *Morus*

## Abstract

Anthocyanins and flavones are important pigments responsible for the coloration of fruits. Mulberry fruit is rich in anthocyanins and flavonols, which have multiple uses in traditional Chinese medicine. The antinociceptive and antibacterial activities of total flavonoids (TF) from black mulberry (MnTF, TF of *Morus nigra*) and non-black mulberry (MmTF, TF of *Morus mongolica*; and MazTF, TF of *Morus alba* ‘Zhenzhubai’) fruits were studied. MnTF was rich in anthocyanins (11.3 mg/g) and flavonols (0.7 mg/g) identified by ultra-performance liquid chromatography–tunable ultraviolet/mass single-quadrupole detection (UPLC–TUV/QDa). Comparatively, MmTF and MazTF had low flavonol contents and MazTF had no anthocyanins. MnTF showed significantly higher antinociceptive and antibacterial activities toward *Escherichia coli*, *Pseudomonas aeruginosa* and *Staphylococcus aureus* than MmTF and MazTF. MnTF inhibited the expression of interleukin 6 (IL-6), inducible nitric oxide synthase (iNOS), phospho-p65 (p-p65) and phospho-IκBα (p-IκBα), and increased interleukin 10 (IL-10). Additionally, mice tests showed that cyanidin-3-*O*-glucoside (C3G), rutin (Ru) and isoquercetin (IQ) were the main active ingredients in the antinociceptive process. Stronger antinociceptive effect of MnTF was correlated with its high content of anthocyanins and flavonols and its inhibitory effects on proinflammatory cytokines, iNOS and nuclear factor-κB (NF-κB) pathway-related proteins.

## 1. Introduction

Mulberry is a deciduous tree or shrub of the genus *Morus* in the family *Moraceae* [1]. It has been cultivated and used in traditional medicine by humans for more than 5000 years [2,3,4]. Mulberry originated in China and is grown throughout Korea, Japan, Mongolia, Southwest Asia, Central Asia, Russia, Europe and South America [5,6]. Eight species of *Morus* were identified by phylogenetic analysis of internal transcribed spacer sequences [7]. The species can also be divided into black mulberry, white mulberry and red mulberry [1].

Previous research has shown that mulberry fruits are rich in anthocyanins, which are responsible for the fruit color [5,8,9]. Anthocyanins have important roles in plants and animals, such as protecting plants from damage caused by UV light, attracting pollinators and serving as antioxidants [10,11,12]. They also have pharmacological activities, including anti-inflammatory, antitumor, and blood lipid-regulating activities [3,13,14,15]. Mulberry fruits also contain many other active substances, such as flavonols, polyphenols, alkaloids and polysaccharides [1,3,8,16]. Modern pharmacological studies have shown that mulberry fruits may provide health benefits through antioxidant, hypoglycemic, antiobesity, anti-inflammatory, analgesic and immunomodulatory effects [16,17,18,19,20].

Inflammatory pain is a very common and important basic pathological process. Pain is a key diagnostic criterion in many acute and chronic medical conditions [21,22]. The inflammatory pathways (such as arachidonic acid metabolic, NF-κB and nitric oxide (NO) pathways) and inflammatory biomarkers (such as IL-6, IL-10 and iNOS) are associated with pain [3,22]. The most common diseases associated with trauma and infection are inflammatory diseases. Mechanical damage, bacterial infections (e.g., *E. coli*, *P. aeruginosa* or *S. aureus*), viral infections and some drugs can cause pain. Steroidal anti-inflammatory drugs, such as dexamethasone (Dex), and non-steroidal antinociceptive drugs, such as aspirin (Asp), are used to combat inflammation and pain. However, they are also associated with significant side effects, such as weight loss and gastrointestinal disorders [3,23,24]. Asp is one of the world’s top three classic drugs and is widely used in analgesic and anti-inflammatory applications. However, millions of people suffer from its side effects every year [25,26]. The most widely used antibiotics, such as penicillin and cefoperazone, have limited recognition of adverse consequences [27,28]. Anthocyanins and flavonols, as natural products, have not yet displayed side effects, which is essential for drug development.

Our previous study had identified nine putative genes involved in anthocyanin and flavonoid biosynthesis in mulberry plants, and anthocyanin content correlated with the expression levels of these genes during the fruit ripening process [29]. Previous studies of ours have found that total flavonoids (TF) of black mulberry possess anti-inflammatory and antioxidant activities that might be correlated with its high anthocyanin content [3,30]. However, the differences in compositions are not clear, nor is it clear whether the TFs (MnTF [total flavonoids of *Morus nigra*], MmTF [total flavonoids of *Morus mongolica*] and MazTF [total flavonoids of *Morus alba* ‘Zhenzhubai’]) have antinociceptive and antibacterial activities, and which compound is the main active ingredient. Therefore, the aim of this study was to compare the compositions and antinociceptive and antibacterial activities of TFs from black and non-black mulberry fruits and to explore the main active ingredients of these effects.

## 2. Results

### 2.1. Determination of Anthocyanin and Flavonol Contents

**Figure 1:**
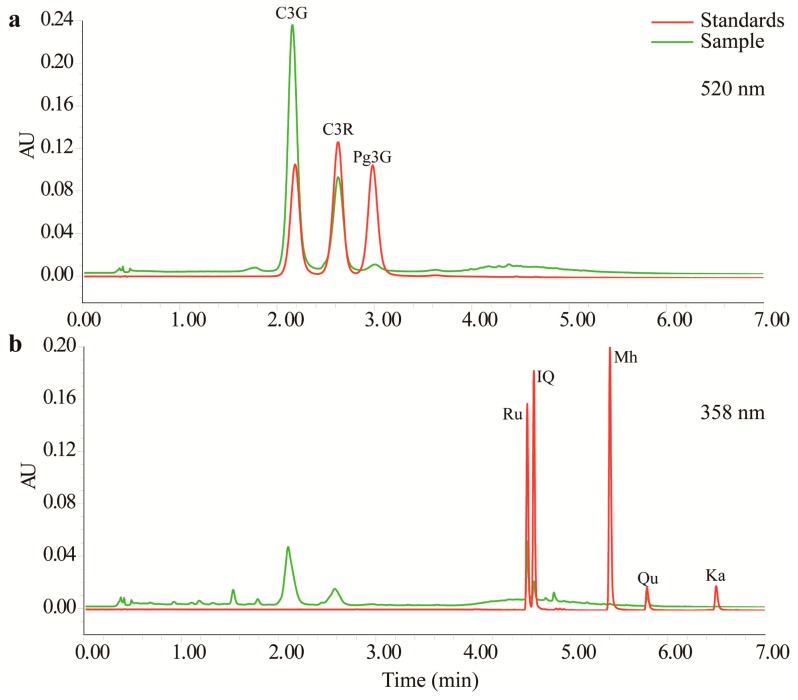
Chromatograms of anthocyanins (**a**) and flavonols (**b**) obtained by UPLC–TUV/QDa.

Three anthocyanins and five flavonols were detected in the TFs by UPLC with tunable ultraviolet (TUV) and quadrupole dalton (QDa) detectors. As shown in Figure 1 and Supplementary Material Figure S1, this method completely resolved all eight compounds within 7 min. As shown in Table 1, all of the calibration curves had good linearity (*r*^2^ > 0.99). MnTF and MmTF contained all eight compounds, while MazTF did not contain any anthocyanins. MnTF had more anthocyanins and flavonols than MmTF and MazTF. The C3G, cyanidin-3-*O*-rutinoside (C3R) and pelargonidin-3-*O*-glucoside (Pg3G) contents of MnTF were 8.2, 2.9 and 0.3 mg/g, respectively. All five flavonols were scarce (<0.5 mg/g), especially morin hydrate (Mh) and kaempferol (Ka), which were present in very low amounts or could not be detected. The flavonol contents of MazTF were lower than those of MnTF and MmTF, except for Qu, which was more abundant in MazTF (0.0036 mg/g) than in MmTF (0.0029 mg/g). In general, black mulberries were rich in anthocyanins and flavonols, while non-black mulberries had low amounts of flavonols and few anthocyanins.

### 2.2. Toxicity Assessment of TFs

**Figure 2:**
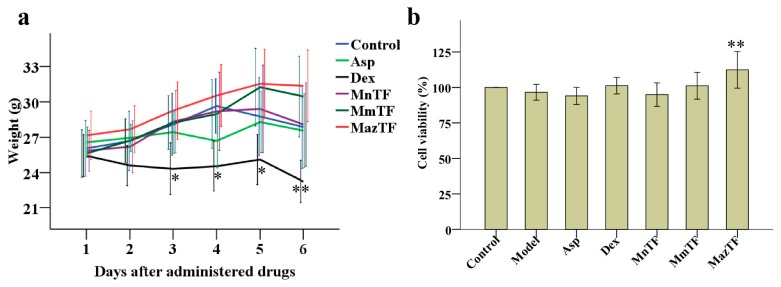
Effects of TFs on weight of mice (**a**) and cytotoxicity of RAW 264.7 cells (**b**). Groups of mice were pretreated (p.o.) with reverse-osmosis water (control and model groups, 20 mL/kg), Asp (aspirin, 150 mg/kg), Dex (dexamethasone, 3 mg/kg), or TFs (5 g crude extract/kg). Data are means ± SD (*n* = 10). RAW 264.7 cells were treated with DMEM (control group), 1 µg/mL lipopolysaccharide (LPS, model group), 1 µg/mL LPS + 0.1 mg/mL Asp, 1 µg/mL LPS + 0.1 mg/mL Dex, or 1 µg/mL LPS + 50 mg crude TF extract/mL. Cell viability is expressed as a percentage of that in the control group, which was set at 100%. Data are means ± SD (*n* = 3).Values with asterisks are significantly different (** *p* < 0.01) from those in the control group in (**a**) or the model group in (**b**).

Changes in weight and cytotoxicity were used to evaluate the toxicity of drugs in vivo and in vitro. The weight of mice decreased gradually after administration of Dex. Side effects were shown in mice when administered at a dose of 1.5 mg/kg Dex. No significant differences in the weight of mice were detected between the control group and groups treated by TFs (MnTF, MmTF and MazTF) at a dose of 5 g crude extract/kg (Figure 2a). The levels of cytotoxicity were assayed by RAW 264.7 cells in vitro. As shown in Figure 2b, none of the drugs were cytotoxic to cells at the administered dose.

### 2.3. Antinociceptive Activities of TFs

**Figure 3:**
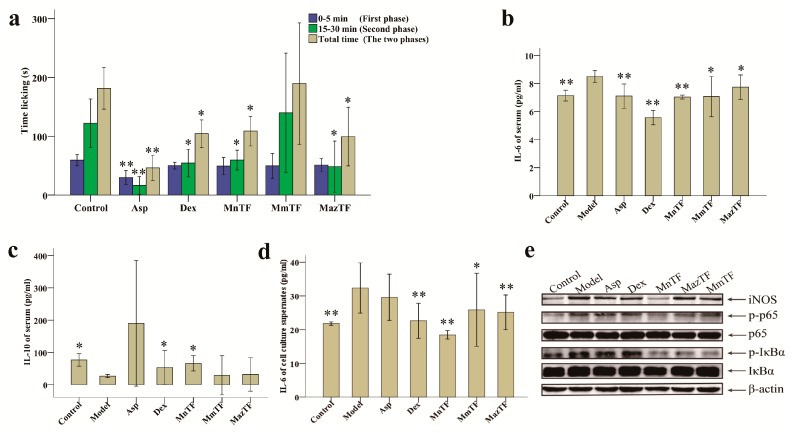
Effects of TFs on pain (**a**); levels of IL-6 (**b**) and IL-10 (**c**) in mice; and levels of IL-6 (**d**) and the expression of pain-related proteins (**e**) in RAW 264.7 cells. Values with asterisks are significantly different (* *p* < 0.05; ** *p* < 0.01) from those in the control group in (**a**); or the model group in (**b**–**d**).

The response pattern in the formalin-induced pain test consists of two phases, a neurogenic pain phase (0–5 min) and an inflammatory pain phase (15–30 min). As shown in Figure 3a and Supplementary Material Table S1, Asp, an antinociceptive drug, significantly reduced the duration of both phases, while Dex significantly reduced the duration of the secondary phase. The secondary phase in the MnTF and MazTF groups (60 ± 20 s and 48 ± 52 s, respectively) was significantly shorter (*p* < 0.05) than that in the control group (122 ± 49 s). MmTF did not show antinociceptive activity, as it did not significantly reduce the licking (licking, biting or flinching) time of either phase. 

### 2.4. Effects of TFs on Cytokines and Pain-Related Proteins

To study the mechanism of antinociceptive effects of TFs, we measured levels of an inflammatory cytokine (IL-6) and an anti-inflammatory cytokine (IL-10). As shown in Figure 3b, the IL-6 level in serum was significantly reduced by MnTF (7.0 pg/mL), MmTF (7.1 pg/mL) and MazTF (7.7 pg/mL) compared with the model group (8.5 pg/mL). There was a similar trend in cell culture supernates (Figure 3d). Only MnTF significantly increased the serum level of IL-10, from 26.7 pg/mL to 66.0 pg/mL (*p* < 0.05), after injury (Figure 3c). In summary, TF of black mulberries significantly reduced the level of an early and mid-term development of inflammatory cytokine (IL-6) and increased the level of an anti-inflammatory cytokine (IL-10), while TF of non-black mulberries had significant effects on IL-6 production.

Western blotting was used to investigate the effects of TFs on the expression of inflammation-related proteins (iNOS, p65, IκBα, p-p65 and p-IκBα) in RAW 264.7 cells. As shown in Figure 3e and Supporting Information Figure S2, the expression levels of p65 and IκBα were not significantly different among groups, while expression of the phosphorylated products (p-p65 and p-IκBα) decreased significantly in the TF-treated groups, especially the MnTF group. The expression level of iNOS was increased in all groups except the MnTF group. Thus, TFs of black mulberries had stronger effects on p-p65, p-IκBα and iNOS expression than TFs of non-black mulberries. A schematic representation of the inhibitory effect of MnTF on the NF-κB and NO pathways is shown in Supplementary Material Figure S3.

### 2.5. Antinociceptive Activities of C3G, Ru and IQ

**Figure 4:**
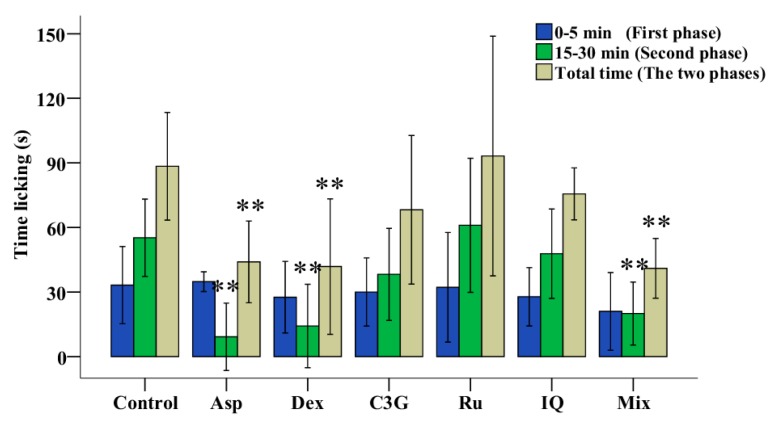
Antinociceptive activities of C3G, Ru and IQ in mice. Values with asterisks are significantly different (* *p* < 0.05; ** *p* < 0.01) from those in the control group.

The mice tests of three main flavonoids of TFs were performed to learn the main active ingredients in the antinociceptive process. As shown in Figure 4 and Supplementary Material Table S1, neither C3G, Ru nor IQ individually reduced the duration of both phases, while the mix (C3G, Ru and IQ) significantly reduced the duration of the secondary phase (inflammatory pain phase). Therefore, anthocyanins and flavonols work together to yield more effective antinociceptive activity. 

### 2.6. Antibacterial Activities of TFs

**Figure 5:**
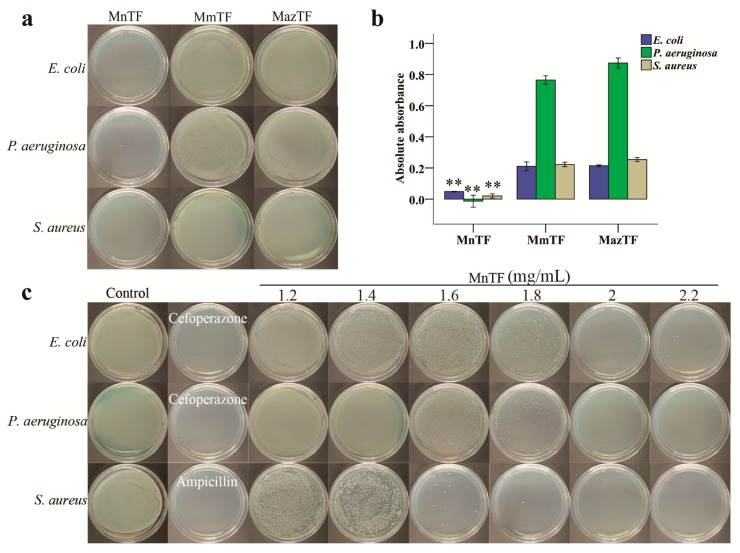
Antibacterial activities of TFs shown on plates (**a**) and by absorbance (**b**); and minimum bactericidal concentration (MBC) of MnTF against *E. coli*, *P. aeruginosa* and *S. aureus* (**c**). The concentration of TFs was 1.8 mg/mL (**a**). Data are means ± SD (*n* = 3). Values with asterisks (**) are significantly different (*p* < 0.01) from those in the MmTF and MazTF groups.

*E. coli*, *P. aeruginosa* and *S. aureus* are three species of inflammatory pain-causing bacteria. As shown in Figure 5a, MnTF strongly inhibited all three bacteria. While bacteria barely grew on Luria–Bertani (LB) plates after being treated with MnTF at 1.8 mg/mL, MmTF-treated and MazTF-treated bacteria covered the plates. Additionally, Figure 5b shows that the antibacterial activity of MnTF was stronger than that of MmTF (*p* < 0.01) or MazTF (*p* < 0.01). In general, TFs of black mulberries had stronger antibacterial activity than TFs of non-black mulberries.

In the minimum bactericidal concentration (MBC) test, MnTF showed strong, dose-dependent antibacterial activities against all three bacteria (Figure 5c). The MBCs of MnTF for *E. coli*, *P. aeruginosa* and *S. aureus* were 2, 2 and 1.8 mg/mL, respectively (Figure 5c and Figure S4).

## 3. Discussion

Mulberry fruits have many bioactive components, such as anthocyanins, flavonols and polysaccharides [3,9,18]. In this study, the chromatography method we used enabled detection of three anthocyanins in mulberry fruits (Figure 1a). The key factor in the UPLC method was the proportion of acetonitrile (ACN) in mobile phase B. The contents of C3G (8.2 mg/g) and C3R (2.9 mg/g) in MnTF were consistent with those in our previous study (8.3 mg/g and 2.9 mg/g, respectively) [3]. However, one other anthocyanin (Pg3G) and four additional important flavonols were identified by UPLC–TUV/QDa in MnTF in this study. Thus, most of the flavonoids identified in mulberry fruits were flavonols. Although five anthocyanins and many flavonols were previously identified in mulberry extracts by high-performance liquid chromatography (HPLC) or UPLC, it was difficult to achieve good resolution of the anthocyanin peaks [5,31]. C3G and C3R are the main anthocyanins responsible for the color of mulberry fruits. The anthocyanin and flavonol contents of black mulberries were about 40-times and 1.3-times higher, respectively, than those of *M. mongolica* fruits (Table 1). 

Weight variation in mice and cytotoxicity are widely used to evaluate the drug toxicity. Our study showed that Dex had significant side effects in mice (Figure 2a), which is consistent with other studies [23,24,32]. In contrast, TFs were not toxic to mice and cells as can be seen by the unchangeable weight and high levels of cell viability similar to the control group (Figure 2a,b). Furthermore, mulberry is a traditional fruit that has long been consumed by humans. Therefore, mulberry TFs are considered to be safe when used for functional development as health products.

Formalin-induced pain-like behavior in mice was a classic model of inflammation and pain [33,34]. Inflammatory pain is divided into a neurogenic phase (initial phase, 0–5 min) and an inflammatory phase (secondary phase, 15–30 min) in the formalin-induced pain model [3,34]. In this study, MnTF and MazTF showed stable antinociceptive effect (Figure 3a,b,d). Moreover, MazTF had low flavonol contents and had no anthocyanins by UPLC–TUV/QDa. This means that flavonols play an important role in the analgesic effect. Some previous studies have also proved this view [35,36,37]. Meanwhile, treatment of mice with MnTF at a dose of 5 g crude extract/kg reduced the duration of the secondary phase (Figure 3a and Table S1). A previous study showed that MnTF at a dose of 2.5 g crude extract/kg could also reduce the duration of the neurogenic phase [3].

Pain and inflammation are related to the arachidonic acid metabolic (AAM) pathway, the NF-κB pathway and the NO pathway [38,39,40]. Dex (a steroidal anti-inflammatory drug) and Asp (a non-steroidal antinociceptive drug) combat pain and inflammation by inhibiting leukotrienes and prostaglandins of the AAM pathway, but they are associated with significant side effects. Previous studies [38,41,42] and this study showed that MnTF regulated the inflammatory process mainly by affecting the NF-κB and NO pathways. The expression of IL-6 is regulated by the NF-κB [43]. NF-κB is normally present in cells in a p50/p65 heterodimeric inhibitory state, which can be activated by phosphorylation, thereby promoting the production of IL-6, TNF-α, IL-1β and so on [44,45,46]. In the early stage of inflammatory infection, IL-6 is induced by TNF-α and keeps a rising trend for a long time in the later period [34,47]. IL-6 may be a feedback inhibition factor of TNF-α and may negatively regulate the production and release of endogenous TNF-α [48,49,50]. As shown in Figure 3e and Supplementary Material Figure S2, MnTF inhibited the expression of iNOS, p-IκBα and p-p65, reduced the levels of pain-related cytokines (TNF-α, IL-1β, IL-6, IFN-γ and NO), and increased the level of an anti-inflammatory cytokine (IL-10). Asp and Dex regulate the inflammatory and antinociceptive process by affecting the AAM pathway. The inhibition of AAM blocked the activity of cyclooxygenase 1 (COX-1), which resulted in the intestinal lesions and the weight changes in mice [40,51]. MnTF did not have this side effect (Figure 2a,b). 

The mixed reagents of C3G, Ru and IQ showed significant antinociceptive activity (Figure 4 and Table S1). Although MazTF had no anthocyanins, containing Ru, IQ and Qu (Table 1), it showed excellent non-toxic and antinociceptive activities (Figure 2 and Figure 3a). Therefore, the fundamental reason why MnTF showed excellent anti-inflammatory activities was that MnTF contains the three main active ingredients, and the flavonols might be the key active substances to play an analgesic effect with anthocyanins, synergistically.

In this study, we investigated the antibacterial properties of anthocyanins and flavonols from mulberry fruits against *E. coli*, *P. aeruginosa* and *S. aureus*. The antibacterial activity of TFs from black mulberries was stronger than that of TFs from non-black mulberries (Figure 5a,b). In the MBC assay, we observed that MnTF showed stronger inhibitory activity against *S. aureus* than against *E. coli* (Figure 5c and Figure S4). Similarly, Wang, Li and Jiang (2010) reported that mulberry polysaccharides had antibacterial activities against *Bacillus subtilis*, *E. coli* and *S. aureus*, and that the antibacterial activity against *E. coli* was especially strong [52]. Morin of mulberry fruits moderately inhibited *Streptococcus mutans*. Moreover, stem bark of *M. alba* var. *alba*, *M. alba* var. *rosa* and *Morus rubra* were potent antimicrobial agents against bacteria that cause infections in humans (*S. aureus*, *Enterococcus faecalis*, *Staphylococcus epidermis*, *E. coli* and *Salmonella Typhimurium bacteria*) [53]. Thus, various parts of mulberry, such as fruits, leaves and stem bark, have antibacterial activities against a variety of bacteria. 

Bacteria cause the oxidative stress reaction and produce reactive oxygen species in the body [54,55]. Polyhydroxy compounds inhibit oxidative stress and induce the body’s release of the related inflammatory factors [56]. Black mulberry is rich in anthocyanins and flavonols, which might be responsible for its antioxidant activity. More in-depth experiments need to be done in the future.

## 4. Materials and Methods

### 4.1. Mulberry Fruits and Animals

Fruits of *M. nigra* were collected at the Hetian Sericultural Research Institute (37°08′50.85″ N, 79°54′26.99″ E; Xinjiang, China) in July 2016. Fruits of *M. mongolica* and *M. alba* ‘Zhenzhubai’ (a mulberry cultivar) were collected from the mulberry breeding center at Southwest University (29°49′36.72″ N, 106°25′29.19″ E; Chongqing, China) in May 2016. The voucher specimens of *M. nigra* (Mn-20160720), *M. mongolica* (Mm-20160515) and *M. alba* ‘Zhenzhubai’ (Maz-20160515) were kept at our laboratory. The fruits were homogenized, oven-dried to a constant mass, and ground into a powder. The powder was sieved with a 60-mesh sieve and stored at −40 °C for further analysis. Male Kunming mice (about 18–22 g) were purchased from Chongqing Medical University, Chongqing. Experiments were carried out according to the guidelines of the International Association for the Study of Pain on the use of animals in pain research. This research was approved by the Animal Care Committee of Southwest University (License number: SCXK (JUN) 2012-0011).

### 4.2. Chemicals and Reagents

C3G, C3R, Pg3G, and LPS were purchased from Sigma-Aldrich (St. Louis, MO, USA). Ru, IQ, Mh, Qu, and Ka were obtained from ChromaBio (Chengdu, China). Phosphoric acid, ACN, and methanol for UPLC were purchased from Thermo Fisher Scientific (Waltham, MA, USA). Asp and Dex for animal experiments were purchased from Original (Shenyang, Liaoning, China) and Xianju Pharma (Hangzhou, Zhejiang, China), respectively. Ultrapure water was prepared using a Milli-Q system (Millipore, Billerica, MA, USA).

The strains of *E. coli* (CMCC44102), *P. aeruginosa* (CMCC10104), and *S. aureus* (CMCC26003) were provided by the National Center for Medical Culture (CMCC) (Beijing, China) and kept at our laboratory. Enzyme-linked immunosorbent assay (ELISA) kits for IL-6 and IL-10 were purchased from CUSABIO (Wuhan, China). Fetal bovine serum, antibiotics (streptomycin and penicillin), trypsin, and DMEM were purchased from Gibco (Grand Island, NY, USA). MTT and BeyoECL Plus and Enhanced BCA Protein Assay kits were purchased from Beyotime (Shanghai, China). Western blotting reagents were purchased from Cell Signaling Technology (Boston, MA, USA).

### 4.3. Extraction of TFs

TFs were extracted and measured as previously described [30] except that the final extraction volume was increased from 200 mL to 400 mL. MnTF, MmTF, and MazTF represent flavonoid extracts of *M. nigra*, *M. mongolica*, and *M. alba* ‘Zhenzhubai’, respectively.

### 4.4. UPLC–TUV/QDa Conditions and Determination of TFs

Chromatographic separation was carried out on a Waters Acquity UPLC I-Class system coupled with TUV and a single-quadrupole mass detector (QDa) with an electrospray ionization source and an Acquity UPLC BEH C_18_ column (1.0 × 100 mm, 1.7 µm, Waters, Milford, MA, USA). The solvent system consisted of a binary mobile phase: solution A was Milli-Q water with 0.2% (*v*/*v*) H_3_PO_4_, and solution B was 40% (*v*/*v*) ACN with 0.2% (*v*/*v*) H_3_PO_4_. The linear elution gradient profile was as follows: 0–3 min, 20–27% B, curve 6; 3–6.5 min, 27–84% B, curve 5; 6.5–7 min, 84–20% B, curve 1. The flow rate was 0.17 mL/min, the column temperature was kept at 40 °C, and the injection volume was 1 µL. The detection wavelengths were 520 nm and 358 nm for anthocyanins and flavonoids, respectively. Concentration detection range: C3G (1.56–100 μg/mL), C3R (0.78–50 μg/mL), P3G, Ru, IQ, Mh, Qu, and Ka (1.56–100 μg/mL).

QDa detector was achieved in SIR and positive electrospray ionization mode (ESI+) mode. The capillary voltage was 0.8 kV and the probe temperature was 600 °C. The sampling frequency was 5 Hz. The 287 *m*/*z* (Ka), 303 *m*/*z* (Ru, IQ, Mh, and Qu), 433 *m*/*z* (P3G), 449 *m*/*z* (C3G), and 595 *m*/*z* (C3R) ions were monitored with a cone voltage of 21 kV.

### 4.5. Evaluation of Toxicity and Antinociceptive Activity in Mice

Male Kunming mice were divided into groups (*n* = 10 for each group) and treated with reverse-osmosis water (20 mL/kg; control), Asp (150 mg/kg), Dex (3 mg/kg), TFs (5 g crude extract/kg), C3G, Ru, or IQ (40 mg/kg), and Mix (Mixed solution of 40 mg/kg C3G, 2.33 mg/kg Ru, and 0.87 mg/kg IQ). The mice were fed adaptively for three days and after that drugs were administered by gavage once per day for seven days. The weights of mice were measured and recorded before gavage and on each of the last six days of drug administration.

Antinociceptive effect was studied in a formalin-induced mouse pain-like behavior model according to previously described methods [3,33,34]. Ten microliters of 2.5% (*v*/*v*) formalin solution was injected into the left hind paw of mice in all groups. Then, the total number of mice that exhibited pain-like behaviors (licking, biting, and flinching) was recorded in the neurogenic phase (initial phase, 0–5 min after formalin injection) and the inflammatory phase (secondary phase, 15–30 min after formalin injection).

### 4.6. Immunological Procedures

#### 4.6.1. Blood Collection for Cytokines Analysis

Male Kunming mice were divided into seven groups and treated: the six groups described in Section 4.5, and a model group was added and treated with reverse-osmosis water (20 mL/kg). After the last treatment of drugs, the dorsal side of the right ear of all mice, except those in the control group, was treated with 0.2 mL of xylene and 0.2 mL of 0.4% (*v*/*v*) acetic acid (intraperitoneal injection). Three hours later, an eyeball was extirpated and blood was collected. Blood samples were clotted overnight at 4 °C and then centrifuged at 1000× *g* for 15 min. The serum was immediately assayed or stored at −40 °C [3].

#### 4.6.2. Cell Culture, Cytotoxicity and Western Blot Analysis

RAW 264.7 cells were cultured in 24-well plates and treated with DMEM (control group), 1 µg/mL LPS (model group), 1 µg/mL LPS + 0.1 mg/mL Asp, 1 µg/mL LPS + 0.1 mg/mL Dex, or 1 µg/mL LPS + 50 mg crude TFs extract/mL. After addition of the drugs, cells were incubated for 24 h and then IL-6 was assessed by ELISA, and cytotoxicity was assessed by the MTT method [38,41]. 

Expression of key regulatory proteins, including iNOS, p65, IκBα, and the phosphorylated products p-p65 and p-IκBα, was analyzed by Western blotting [42,57]. RAW 264.7 cell lysates (25 μg of protein) were subjected to electrophoresis on 12% sodium dodecyl sulfate–polyacrylamide gels. The resolved proteins were transferred to polyvinylidene fluoride membranes (240 mA for 90 min). The membranes were incubated with Tris-buffered saline (TBS) containing 5% (*w*/*v*) nonfat milk at 4 °C overnight, washed with TBS–Tween 20 (TBST) for 5 min, incubated with specific antibodies against iNOS, p65, IκBα, p-p65, p-IκBα, or β-actin for 1 h, and washed three times with TBST for 10 min. Then, the membranes were incubated with horseradish peroxidase-conjugated secondary antibody for 1 h and washed three times with TBST for 10 min. Blots were visualized using the BeyoECL Plus kit.

### 4.7. Antibacterial Assays

#### 4.7.1. Comparison of Antibacterial Activities

Three bacteria (*E. coli*, *P. aeruginosa*, and *S. aureus*) were used to evaluate antibacterial activity of TFs. Briefly, 90 mg/mL TFs was diluted 50 fold by LB medium (the effective concentrations of C3G, Ru and Qu were 6.4 μg/mL, 0.8 μg/mL and 0.4 μg/mL, respectively). 0.2 mL of the diluted TFs was mixed with 0.02 mL of bacterial suspension (OD_600_ = 0.6) and incubated at 37 °C for 24 h. The antibacterial activity was evaluated by measuring the OD_600_ of the bacterial suspension. Then, 0.1 mL of the culture was spread on LB plates. The inoculated plates were incubated at 37 °C for 24 h and then the numbers of colonies were recorded.

#### 4.7.2. MBC of MnTF

MBC is the minimum drug concentration required to kill 99.9% of the test microorganisms or inhibit the growth of colonies to not more than five. For *E. coli*, *P. aeruginosa*, and *S. aureus*, the positive control drugs were cefoperazone (100 μg/mL), cefoperazone (200 μg/mL), and ampicillin (100 μg/mL), respectively. Six isocratic solutions of MnTF (60 mg/mL to 110 mg/mL, MnTF was diluted 50 fold by LB medium) were prepared. Then, 0.2 mL of drug or MnTF was mixed with 0.02 mL of bacterial suspension (OD_600_ = 0.6) and incubated at 37 °C for 24 h. The OD_600_ of the bacterial suspension was measured and then 0.1 mL of the culture was spread on LB plates. The inoculated plates were incubated at 37 °C for 24 h and then the numbers of colonies were recorded [58].

### 4.8. Statistical Analyses

Results were expressed as means ± standard deviation (SD). Data were analyzed using SPSS Statistics version 17.0. One-way analysis of variance was used for intergroup comparisons; *p* values < 0.05 were considered statistically significant.

## 5. Conclusions

Three anthocyanins and five flavonols were identified in mulberry fruits. TF of black mulberry exhibited stronger antinociceptive and antibacterial effects than that of non-black mulberries. One of the conclusions made from the present study demonstrated that anthocyanins (C3G) and flavonols (Ru and IQ) were responsible for, or at least would be correlated with, the antinociceptive effect of black mulberry. Evidence of inhibitory effects on proinflammatory cytokines, iNOS and NF-κB pathway-related proteins underlying antinociceptive and antibacterial effects of mulberry TF will expand our knowledge of anthocyanins and flavonols, and could be incorporated into alternatives for analgesic and antibacterial drugs. 

## Figures and Tables

**Table 1:** Information of chromatography and MS of *M. nigra*, *M. mongolica* and *M. alba* ‘Zhenzhubai’.

Compounds	RT ^*a*^ (min)	Regression Equation ^*b*^	*r*^2^	Content (mg/g) ^*c*^			Selected Ions by QDa (*m*/*z*)
				MnTF	MmTF	MazTF	
C3G	2.186	*y* = (12.499*x* + 1.239) × 10^3^	0.9999	8.2168 ± 0.0238	0.2220 ± 0.0024	ND	449.18
C3R	2.627	*y* = (8.765*x* + 1.550) × 10^3^	0.9999	2.8578 ± 0.0146	0.0610 ± 0.0013	ND	595.33
P3G	2.983	*y* = (5.230*x* + 0.770) × 10^3^	0.9999	0.2539 ± 0.0047	0.0057 ± 0.0003	ND	433.24
Ru	4.556	*y* = (6.065*x* + 2.362) × 10^3^	0.9999	0.4498 ± 0.0075	0.2723 ± 0.0013	0.0816 ± 0.0015	302.93
IQ	4.624	*y* = (2.560*x* + 0.080) × 10^3^	0.9999	0.1639 ± 0.0006	0.2459 ± 0.0059	0.0631 ± 0.0033	303.06
Mh	5.405	*y* = (6.880*x* + 0.226) × 10^3^	0.9999	0.0002 ± 0.0001	<0.0001	<0.0001	303.04
Qu	5.786	*y* = (4.870*x* − 0.074) × 10^3^	0.9993	0.0716 ± 0.0045	0.0029 ± 0.0002	0.0036 ± 0.0004	303.11
Ka	6.497	*y* = (2.710*x* + 0.487) × 10^3^	0.9986	<0.0001	<0.0001	ND	287.03

^*a*^ RT, retention time; *^b^ y*, peak area; *x*, concentration injected (μg/mL); ^*c*^ mg/g, weight of the dry powder; ND, not detected.

## Supplementary Materials

The following are available online, Figure S1: Spectra of anthocyanins and flavonols by UPLC-TUV/QDa; Figure S2. Western blotting of TFs on the expression of inflammation-related proteins. The grayscale of β-actin is set to 1, and the other groups use it as a reference; Figure S3: Schematic representation of the inhibitory effect of MnTF on the cytokines, NF-κB, and NO pathways in RAW 264.7 cells; Figure S4: MBC of MnTF against *E. coli* (a), *P. aeruginosa* (b), and *S. aureus* (c) shown by absorbance. Data are means ± SD (*n* = 3). Values with asterisks (**) are significantly different (*p* < 0.01) from values in the control group; Table S1: Data of antinociceptive activities in mice.

## Abbreviations

ACN	acetonitrile
Asp	aspirin
C3G	cyanidin-3-*O*-glucoside
C3R	cyanidin-3-*O*-rutinoside
Dex	dexamethasone
ELISA	enzyme-linked immunosorbent assay
HPLC	high-performance liquid chromatography
iNOS	inducible nitric oxide synthase
IQ	isoquercetin
IFN-γ	interfron γ
IL-6	interleukin 6
IL-10	interleukin 10
Ka	kaempferol
LPS	lipopolysaccharide
MazTF	total flavonoid of *Morus alba* ‘Zhenzhubai’
MBC	minimum bactericidal concentration
MnTF	total flavonoid of *Morus nigra*
MmTF	total flavonoid of *Morus mongolica*
MeOH	methanol
Mh	morin hydrate
NF-κB	nuclear factor-κB
NO	nitric oxide
p-IκBα	phospho-IκBα
p-p65	phospho-p65
Pg3G	pelargonidin-3-*O*-glucoside
Qu	quercetin
Ru	quercetin-3-*O*-rutinlside
SD	standard deviation
TF	total flavonoids
TFs	total flavonoids of *M. nigra*, *M. mongolica*, and *M. alba* ‘Zhenzhubai’
TNF-α	tumor necrosis factor α
UPLC	ultra-performance liquid chromatographic
UPLC-TUV/QDa	ultra-performance liquid chromatography–tunable ultraviolet/mass single-quadrupole detector
