# Supplementary material for: Antinociceptive and Antibacterial Properties of Anthocyanins and Flavonols from Fruits of Black and Non-Black Mulberries
Source: Molecules. 2017 Dec 21;23(1):4. doi: 10.3390/molecules23010004 (PMC5943937; doi:10.3390/molecules23010004)

molecules

MDPI

**Supplementary Materials:**

**Table S1. Data of antinociceptive activities in mice.**

| | | Control | Asp | Dex | MnTF | MmTF | MazTF | |
|---|---|---|---|---|---|---|---|---|
| Antinociceptive activities of MnTF, MmTF, and MazTF | | (s) | | | | | | |
| | 0-5 min | 59±12 | 30±14 | 50±7 | 49±17 | 50±25 | 51±13 | |
| | 15-30 min | 122±49 | 16±18 | 54±28 | 60±20 | 140±121 | 48±52 | |
| | Total time | 181±42 | 46±25 | 104±28 | 109±30 | 190±123 | 99±60 | |
| | | Control | Asp | Dex | C3G | Ru | IQ | Mix |
| Antinociceptive activities of C3G, Ru, and IQ | | (s) | | | | | | |
| | 0-5 min | 33±14 | 35±4 | 28±13 | 30±13 | 32±20 | 28±11 | 21±15 |
| | 15-30 min | 55±14 | 9±13 | 14±16 | 38±17 | 61±25 | 48±17 | 20±12 |
| | Total time | 88±20 | 44±15 | 42±25 | 68±28 | 93±45 | 76±10 | 41±11 |

**Figure S1: Spectra of anthocyanins and flavonols by UPLC-TUV/QDa.**

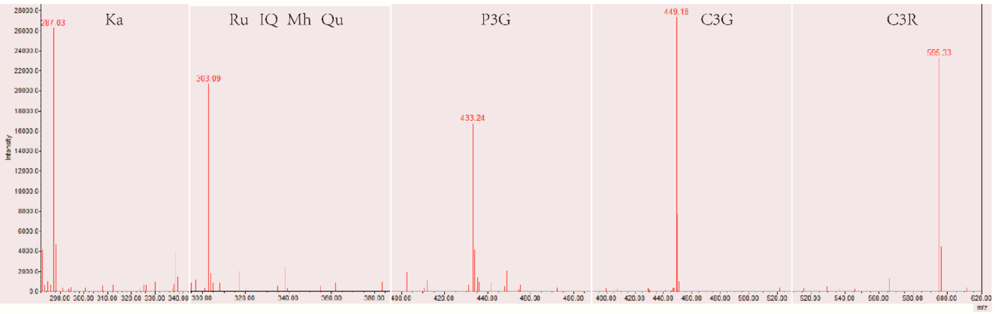


**Figure S2. Western blotting of TFs on the expression of inflammation-related proteins. The grayscale of β-actin is set to 1, and the other groups use it as a reference.**

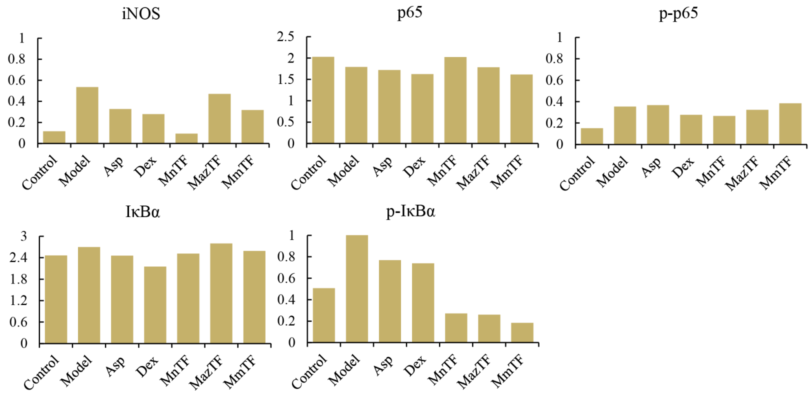


**Figure S3: Schematic representation of the inhibitory effect of MnTF on the cytokines, NF-κB, and NO pathways in RAW 264.7 cells.**

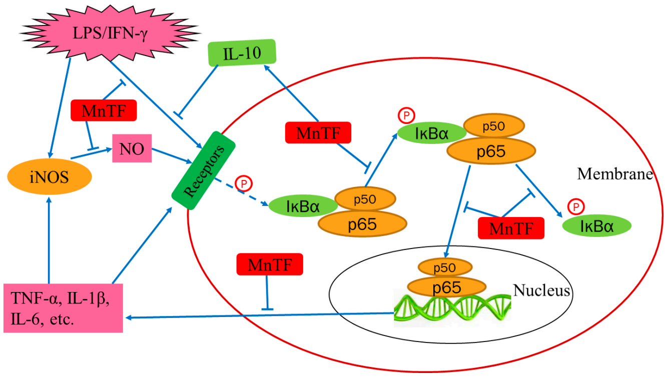


**Figure S4: MBC of MnTF against *E. coli* (a), *P. aeruginosa* (b), and *S. aureus* (c) shown by absorbance. Data are means ± SD (n = 3). Values with asterisks (**) are significantly different ($p$ < 0.01) from values in the control group.**

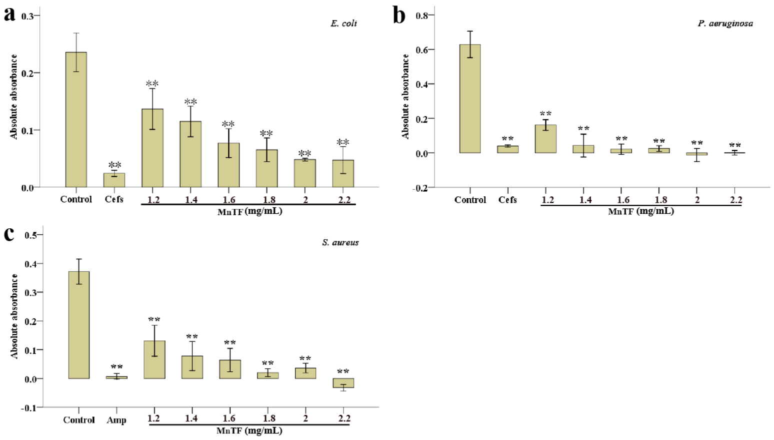

Supplement: Supplementary file 1 [file molecules-23-00004-s001.pdf]
